# RNA sequencing reveals dynamic expression of lncRNAs and mRNAs in caprine endometrial epithelial cells induced by *Neospora caninum* infection

**DOI:** 10.1186/s13071-022-05405-5

**Published:** 2022-08-24

**Authors:** Shan-Shan Zhao, De-Liang Tao, Jin-Ming Chen, Jiang-Ping Wu, Xin Yang, Jun-Ke Song, Xing-Quan Zhu, Guang-Hui Zhao

**Affiliations:** 1 College of Veterinary Medicine, Northwest A&F University, Yangling, 712100 Shaanxi China; 2 College of Veterinary Medicine, Shanxi Agricultural University, Taigu, 030801 Shanxi China; 3 Key Laboratory of Veterinary Public Health of Higher Education of Yunnan Province, College of Veterinary Medicine, Yunnan Agricultural University, Kunming, 650201 Yunnan China

**Keywords:** *Neospora caninum*, Caprine endometrial epithelial cells, Expressed profiles, LncRNAs, mRNAs

## Abstract

**Background:**

The effective transmission mode of *Neospora caninum*, with infection leading to reproductive failure in ruminants, is vertical transmission. The uterus is an important reproductive organ that forms the maternal–fetal interface. *Neospora caninum* can successfully invade and proliferate in the uterus, but the molecular mechanisms underlying epithelial-pathogen interactions remain unclear. Accumulating evidence suggests that host long noncoding RNAs (lncRNAs) play important roles in cellular molecular regulatory networks, with reports that these RNA molecules are closely related to the pathogenesis of apicomplexan parasites. However, the expression profiles of host lncRNAs during *N. caninum* infection has not been reported.

**Methods:**

RNA sequencing (RNA-seq) analysis was used to investigate the expression profiles of messenger RNAs (mRNAs) and lncRNAs in caprine endometrial epithelial cells (EECs) infected with *N. caninum* for 24 h (TZ_24h) and 48 h (TZ_48 h), and the potential functions of differentially expressed (DE) lncRNAs were predicted by using Gene Ontology (GO) enrichment and Kyoto Encyclopedia of Genes and Genomes (KEGG) analysis of their mRNA targets.

**Results:**

RNA-seq analysis identified 1280.15 M clean reads in 12 RNA samples, including six samples infected with *N. caninum* for 24 h (TZ1_24h-TZ3_24h) and 48 h (TZ1_48h-TZ3_48h), and six corresponding control samples (C1_24h-C3_24h and C1_48h-C3_48h). Within the categories TZ_24h-vs-C_24h, TZ_48h-vs-C_48h and TZ_48h-vs-TZ_24h, there were 934 (665 upregulated and 269 downregulated), 1238 (785 upregulated and 453 downregulated) and 489 (252 upregulated and 237 downregulated) DEmRNAs, respectively. GO enrichment and KEGG analysis revealed that these DEmRNAs were mainly involved in the regulation of host immune response (e.g. TNF signaling pathway, MAPK signaling pathway, transforming growth factor beta signaling pathway, AMPK signaling pathway, Toll-like receptor signaling pathway, NOD-like receptor signaling pathway), signaling molecules and interaction (e.g. cytokine-cytokine receptor interaction, cell adhesion molecules and ECM-receptor interaction). A total of 88 (59 upregulated and 29 downregulated), 129 (80 upregulated and 49 downregulated) and 32 (20 upregulated and 12 downregulated) DElncRNAs were found within the categories TZ_24h-vs-C_24h, TZ_48h-vs-C_48h and TZ_48h-vs-TZ_24h, respectively. Functional prediction indicated that these DElncRNAs would be involved in signal transduction (e.g. MAPK signaling pathway, PPAR signaling pathway, ErbB signaling pathway, calcium signaling pathway), neural transmission (e.g. GABAergic synapse, serotonergic synapse, cholinergic synapse), metabolism processes (e.g. glycosphingolipid biosynthesis-lacto and neolacto series, glycosaminoglycan biosynthesis-heparan sulfate/heparin) and signaling molecules and interaction (e.g. cytokine-cytokine receptor interaction, cell adhesion molecules and ECM-receptor interaction).

**Conclusions:**

This is the first investigation of global gene expression profiles of lncRNAs during *N. caninum* infection. The results provide valuable information for further studies of the roles of lncRNAs during *N. caninum* infection.

**Graphical Abstract:**

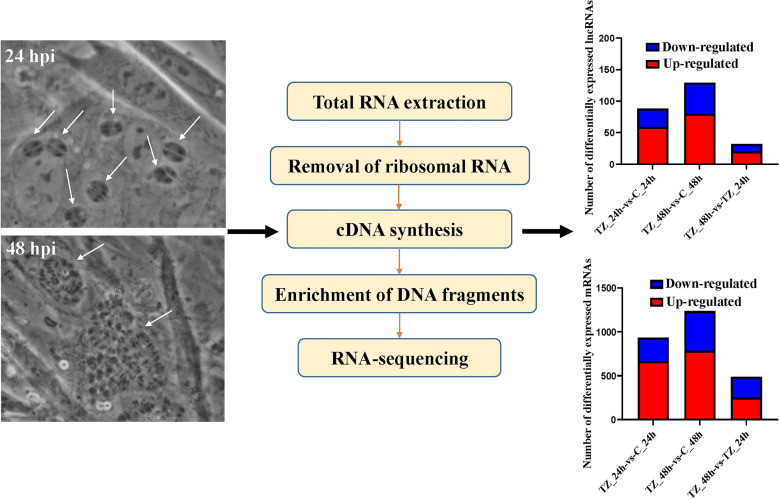

**Supplementary Information:**

The online version contains supplementary material available at 10.1186/s13071-022-05405-5.

## Background

*Neospora caninum*, an obligate intracellular protozoan parasite similar to *Toxoplasma gondii* in morphological and biological features [1, 2], causes serious neurological disorders in canids (e.g. dogs, gray wolves and coyotes) and reproductive failure in cattle and small ruminants (e.g. goats) [3, 4]. Notably, seropositivity against *N. caninum* infection has also been reported in humans, especially in those with immunodeficiency and neurological disorders [5–7]. However, no effective drugs or vaccines against *N. caninum* infection are yet available.

In recent decades, transcriptomics has become an attractive tool for developing new diagnostic or therapeutic targets for the treatment of tumors and infectious diseases through identifying genes of interest or biological events under defined conditions or disease states [8–11]. Transcriptome analysis of bovine trophoblast cells showed a clear effect on extracellular matrix re-organization, cholesterol biosynthesis and the transcription factor AP-1 network by both Nc-Spain1H and Nc-Spain7, two *N. caninum* isolates with significantly different virulences [12]. RNA-sequencing (RNA-seq) of bovine monocyte-derived macrophages (boMØs) showed that genes involved in inflammation, chemokine signaling, cell survival and inhibition of genes related to metabolism and phagolysosome formation were upregulated. Different expression patterns of some genes encoding inflammatory cytokines (e.g. *IL12A*, *IL8* and *IL23*) were also found in boMØs infected with these two isolates [13]. Additionally, *N. caninum* infection induced significantly differentially expressed (DE) genes involved in immune response and lipid biosynthetic processes in rat brain microvascular endothelial cells (rBMVECs), human brain microvascular endothelial cells (hBMECs) [14] and mouse brain samples [15].

Long noncoding RNAs (lncRNAs), a class of RNA transcripts that are larger than 200 nucleotides (nt), exert biological functions by interacting with proteins, DNA or other RNAs at epigenetic, transcriptional and post-transcriptional levels [16, 17]. Studies have shown that dysregulation of the expression of lncRNAs occurs in a large number of diseases, including viral infections (e.g. Epstein-Barr virus, severe acute respiratory syndrome coronavirus 2 and hepatitis B virus) [18–20], bacterial infections (e.g. *Pseudomonas aeruginosa*, *Helicobacter pylori* and *Mycobacteria tuberculosis*) [21–23] and parasitic infections (e.g.* T. gondii*, *Cryptosporidium parvum* and *Eimeria necatrix*) [24–26]. These same studies and others have also shown that differentially expressed (DE) lncRNAs (DElncRNAs) were involved in several key biological processes (e.g. apoptosis, pyroptosis, cell proliferation and metabolism) [17, 27] or implicated in host–pathogen interactions (e.g. promoting or inhibiting pathogenic microorganisms) [28, 29].

The uterus is indispensable for constitution of fetal-maternal interface, embryo implantation and maintenance of pregnancy [30]. The endometrium is particularly susceptible to microbial infections, increasing the risk of adverse pregnancy outcomes [31]. *Neospora caninum* tachyzoites have been detected in the uterus of naturally infected animals, and *N. caninum* tissue cysts have also been found in the endometrium and the maternal–fetal interface (crypt) [32–34]. The objectives of the present study were to investigate the expression profiles of lncRNAs and messenger RNAs (mRNAs) in caprine endometrial epithelial cells (EECs) during *N. caninum* infection.

## Methods

### Parasites, cell cultures and in vitro infection model

*Neospora caninum* Nc-1 wild-type strain was obtained from Prof. Qun Liu (China Agricultural University, Beijing, China) and maintained in African green monkey kidney epithelial cells (Vero cells) provided as a gift by Prof. Xuefeng Qi (Northwest A&F University, Shaanxi, China). An in vitro infection model for *N. caninum* tachyzoites was established as described previously [35] by using caprine EECs supplied by Prof. Yaping Jin (Northwest A&F University, Shaanxi, China).

### Sample collection

Caprine EECs in the experimental groups were infected with 1.2 × 10^6^ freshly egressed *N. caninum* tachyzoites at a multiplicity of infection (MOI) of 3:1 (parasite:cell) for 24 h (experimental groups: TZ1_24h to TZ3_24h) or 48 h (experimental groups: TZ1_48h to TZ3_48h). The caprine EECs without infection of tachyzoites were collected as control groups at 24 h (controls groups: C1_24h to C3_24h) or 48 h (control groups: C1_48h to C3_48h) post-infection (hpi). Cells in all experimental and control groups were collected into TRIzol (Accurate Biotechnology Co., Ltd., Hunan, China) and stored at — 80 °C until RNA extraction.

### RNA extraction, library preparation and RNA-seq

Total RNA samples were extracted from each sample by using a mirVana miRNA Isolation Kit (Ambion, Austin, TX, USA) following the manufacturer’s instructions. The concentration and RNA integrity of the total RNA samples were assessed using the NanoDrop spectrophotometer (Thermo Fisher Scientific, Wilmington, MA, USA) and the Agilent 2100 Bioanalyzer (Agilent Technologies, Santa Clara, CA, USA). RNA samples with a 28S:18S ratio ≥ 0.7 and RNA integrity number ≥ 7 were used for further analysis. The RNA-seq libraries were produced by using TruSeq Stranded Total RNA with Ribo-Zero Gold (Illumina Inc, San Diego, CA, USA) and sequenced using the Illumina sequencing platform (HiSeq^TM^ 2500; Illumina Inc., San Diego, CA, USA). All of these experiments were performed in the laboratory of Shanghai OE Biomedical Science and Technology Company (Shanghai, China).

### Data processing and reference genome mapping

The raw reads obtained by RNA-seq were processed by using SortMeRNA software [36] to remove ribosomal RNA (rRNA) sequences, and reads with low-quality were filtered by using Trimmomatic software [37]. Fastp software [38] was then used to assess the quality of filtered reads through setting a number of important parameters, such as length distribution, Q30 and GC contents. The validated reads (clean reads) were mapped against the reference genome database (ftp://ftp.ncbi.nlm.nih.gov/genomes/all/GCF/001/704/415/GCF_001704415.1_ARS1/GCF_001704415.1_ARS1_genomic.fna.gz) using the Hisat2 (v2.2.1.0) algorithm [39].

### Identification of lncRNAs

Clean reads aligned to the reference genome were assembled by using Stringtie software (v1.3.3b) [40]. The new transcripts with known coding or known loci were filtered out by comparing merged transcripts to reference transcripts. The transcripts with lengths > 200 nt and at least two exons were selected and then predicted for coding potential by using the softwares Pfam (v30) [41], coding-non-coding index (CNCI, 1.0) [42], coding potential calculator 2 (CPC2, beta) [43] and predictor of long non-coding RNAs and messenger RNAs based on an improved *k*-mer scheme (PLEK) [44] to obtain candidate lncRNAs. Known lncRNAs in these candidate lncRNAs were identified by alignment with available lncRNA databases, and unaligned candidates were referred as novel lncRNAs.

### Differential expression analysis of lncRNAs and mRNAs

Expression of lncRNAs and mRNAs were analyzed by using eXpress software [45] to obtain fragments per kilobase of exon per million fragments mapped (FPKM) and count values (the number of reads between specific transcript regions). The DESEQ package (v1.18.0) was used to normalize the counts and calculate the differences in expression by comparing the *P* values and the fold change (FC). The *P* values were then adjusted by using the Benjamini and Hochberg method; genes with *q*-value or false discovery rate (FDR) < 0.05 and Log2|FC| > 1 were considered to be DE genes.

### Target prediction of lncRNAs and functional analysis

The co-expression analysis between DElncRNAs (length < 6000 nt) and differentially expressed mRNAs (DEmRNAs) were conducted based on an absolute values of Pearson’s correlation coefficient ≥ 0.8 and *P* ≤ 0.05. Based on co-expression nets, targets for both *cis* and *trans* regulation were predicted for DElncRNAs. *Cis* targets were searched for all coding genes within 100 kb upstream or downstream of the DElncRNAs by using FEELnc software [46], while *trans* targets of DElncRNAs were screened with the number of direct complementary base pairs ≥ 10 and the base binding free energy ≤ - 100 by using RIsearch-2.0 software [47].

Functions of DElncRNAs were predicted through annotation of targets for both *cis* and *trans* regulation by using Gene Ontology (GO) (http://geneontology.org/) and the Kyoto Encyclopedia of Genes and Genomes (KEGG) (http://www.genome.jp/kegg/) within the Swiss-Prot database (http://www.gpmaw.com/html/swiss-prot.html) and KAAS database (http://www.genome.jp/tools/kaas/), respectively. The significance of GO terms and KEGG pathways enriched were evaluated by using the hypergeometric distribution test, with *q* < 0.05 considered to indicate significance.

### Quantitative real-time PCR analysis

A total of 12 caprine EEC samples with (experimental group) or without (control group) *N. caninum* tachyzoites infection for 24 h or 48 h were collected to verify the accuracy of RNA-seq data by quantitative real-time PCR (qRT-PCR). Total RNA samples were extracted using TRIzol reagent and reversely transcribed to complementary DNA (cDNA) by using the *EVO M-MLV* RT Kit with gDNA Clean for qPCR II (Accurate Biotechnology Co., Ltd, Hunan, China) according to the manufacturer’s instructions. qRT-PCR reactions were performed by using 2 × Universal SYBR Green Fast qPCR Mix (ABclonal, Wuhan, China). The primer sequences designed using DNAMAN 7.0 software (Lynnon Biosoft, Quebec City, QC, Canada) are listed in Additional file 1: Data S1. The glyceraldehyde-3-phosphate dehydrogenase gene (*GAPDH*) was used as an internal reaction control, and three replicate assays were carried out for each gene. The relative expression of each gene was calculated by using the 2 ^−ΔΔCt^ method.

### Statistical analysis

Relative expression levels of selected genes between the experimental and control groups were analyzed by using GraphPad PRISM 8.0.1 software (GraphPad Software Inc., San Diego, CA, USA), and a *P* value < 0.05 was considered to be statistically significant by using two-tailed t-test, with a parametric test.

## Results

### Identification of lncRNAs

**Fig. 1:**
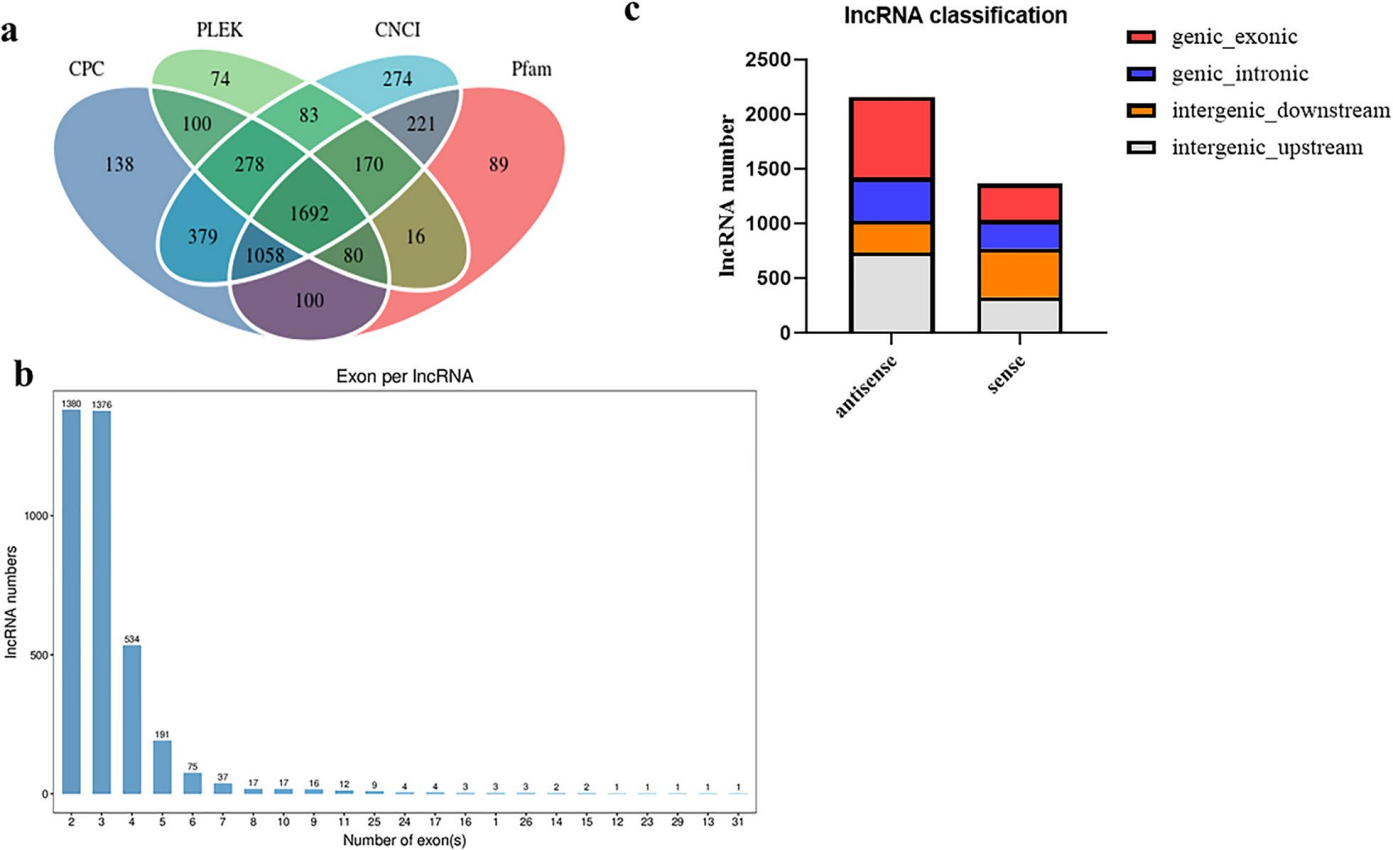
Venn diagrams, exon numbers and classification of candidate long noncoding RNAs (lncRNAs) in caprine endometrial epithelial cells (EECs) following *Neospora caninum* infection. **a** Venn diagrams of coding potential analysis by using Pfam, coding-non-coding index (CNCI), coding potential calculator 2 (CPC2) and predictor of long noncoding RNAs and messenger RNAs based on an improved *k*-mer scheme (PLEK) software programs. **b** Exon numbers of lncRNAs. **c** Classification of lncRNAs by sense and antisense types

In the present study, we generated a total of 1,306.87 M raw reads from 12 samples by using RNA-seq, of which 1280.15 M clean reads were obtained after removing the low-quality reads. The valid bases, quality score (Q30) and average GC contents of these clean reads were 95.43–96.43%, 90.40–92.82% and 48.30%, respectively (Additional file 2: Data S2). Screening using the Pfam, CNCI, CPC2 and PLEK software programs resulted in the identification of 3690 lncRNAs, including 491 novel and 3199 known lncRNAs. The total length of these lncRNAs was 6,253,838 nt and the average length was 1694.81 nt. The number of exons in most of these lncRNAs ranged from 2 to 5 (Fig. 1a, b; Additional file 3: Data S3). Four classifications were identified for these lncRNAs by both antisense and sense types: (i) genic exonic (334 antisense and 291 sense); (ii) genic intronic (395 antisense and 261 sense); (iii) intergenic downstream (291 antisense and 441 sense); and (iv) intergenic upstream (736 antisense and 331 sense) (Fig. 1c; Additional file 3: Data S3).

### DE profiles of lncRNAs and mRNAs

**Fig. 2:**
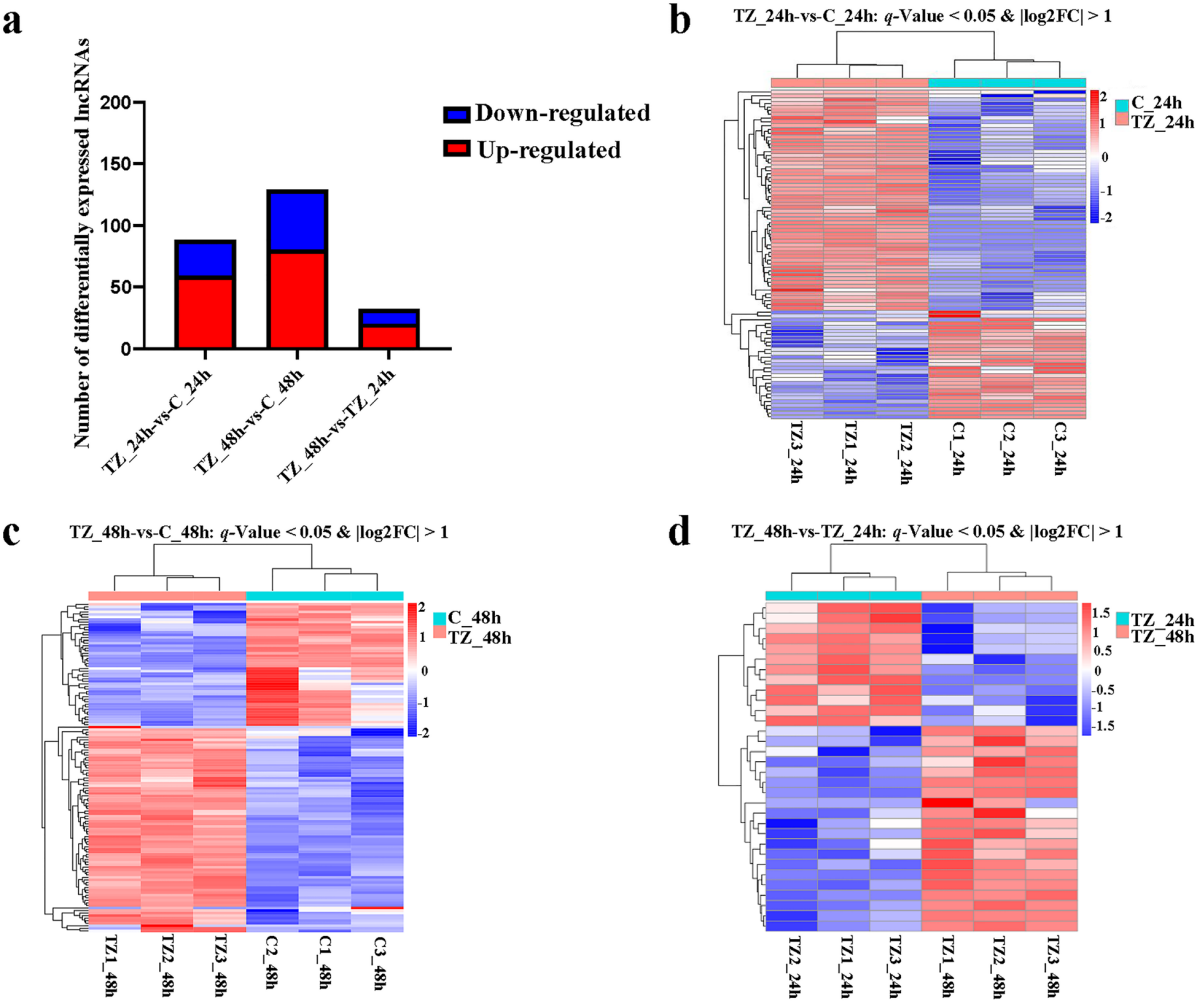
Differentially expressed lncRNAs (DElncRNAs) and hierarchical clustering heatmaps of the DElncRNAs in caprine endometrial epithelial cells (EECs) following *Neospora caninum* infection. **a** The number of DElncRNAs. **b**–**d** Hierarchical clustering heatmaps of the DElncRNAs within the categories TZ_24 h versus C_24 h (TZ_24h-vs-C_24h) (**b**), TZ_48 h versus C_48 h (TZ_48h-vs-C_48h) (**c**) and TZ_48 h versus TZ_24 h (TZ_48h-vs-TZ_24h) (**d**), respectively. *P* values were adjusted by using the Benjamini and Hochberg method, with *q*-value < 0.05 and Log2|FC|> 1 considered to be significant

**Fig. 3:**
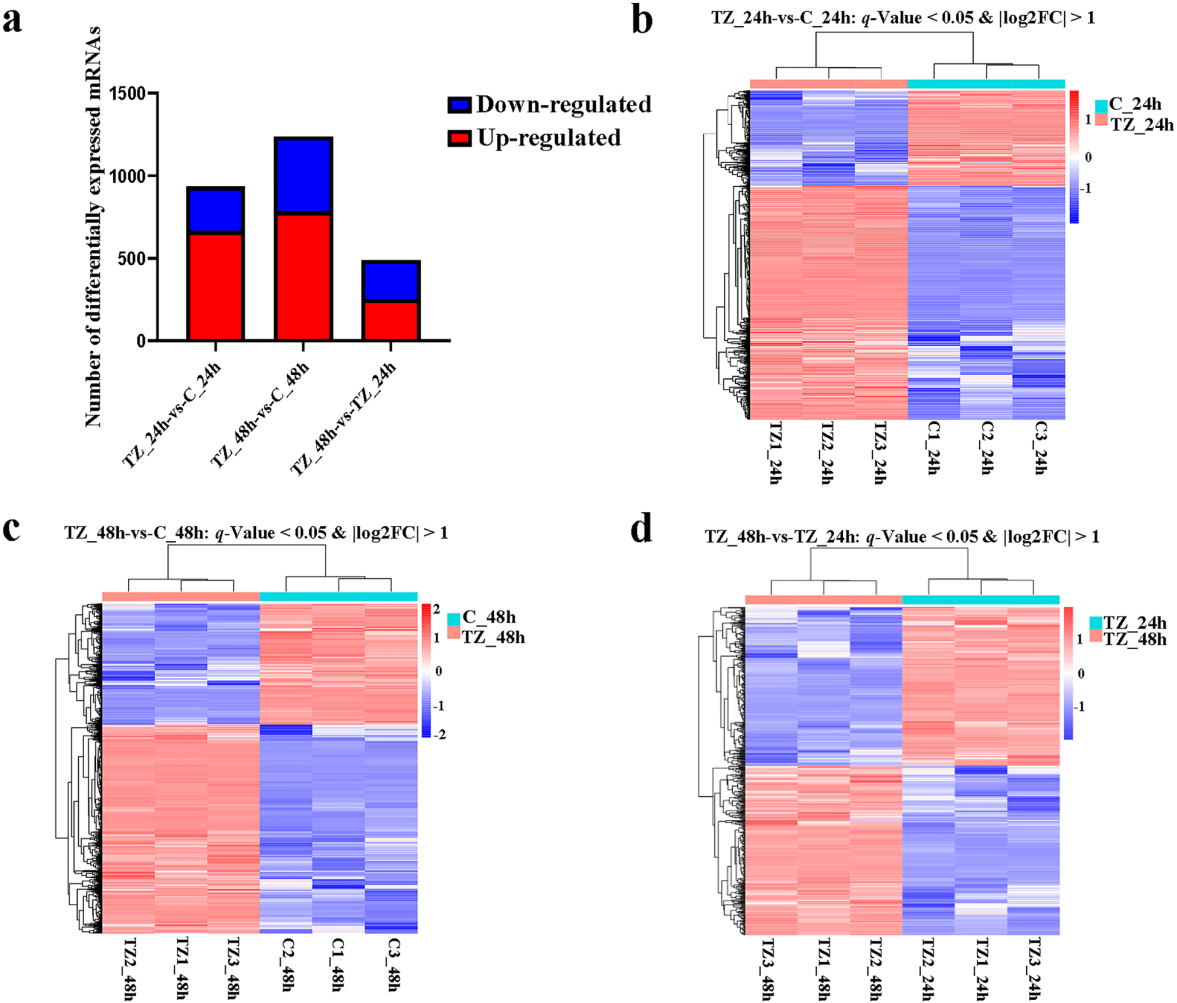
Differentially expressed messenger RNAs (DEmRNAs) and hierarchical clustering heatmaps of the DEmRNAs in caprine endometrial epithelial cells (EECs) following *Neospora caninum* infection. **a** The number of DEmRNAs. **b**–**d** Hierarchical clustering heatmaps of the DEmRNAs within the categories TZ_24h-vs-C_24h (**b**), TZ_48h-vs-C_48h (**c**) and TZ_48h-vs-TZ_24h (**d**), respectively. *P* values were adjusted by using Benjamini & Hochberg method, with *q*-value < 0.05 and Log2|FC|> 1 considered to be significant

To analyze DE profiles of lncRNAs and mRNAs, 12 samples were divided into three categories: (i) TZ_24 h versus C_24 h (TZ_24h-vs-C_24h); (ii) TZ_48 h versus C_48 h (TZ_48h-vs-C_48h); and (iii) TZ_48 h versus TZ_24 h (TZ_48h-vs-TZ_24h). The number of DElncRNAs in the TZ_24h-vs-C_24h, TZ_48h-vs-C_48h and TZ_48h-vs-TZ_24h categories was 88 (59 upregulated and 29 downregulated), 129 (80 upregulated and 49 downregulated) and 32 (20 upregulated and 12 downregulated), respectively (Fig. 2a; Additional file 4: Data S4). The number of DEmRNAs in the categories TZ_24h-vs-C_24h, TZ_48h-vs-C_48h and TZ_48h-vs-TZ_24h was 934 (665 upregulated and 269 downregulated), 1238 (785 upregulated and 453 downregulated) and 489 (252 upregulated and 237 downregulated), respectively (Fig. 3a; Additional file 5: Data S5). In addition, hierarchical clustering heatmaps of the DElncRNAs (length < 6000 nt) (Fig. 2b–d) and DEmRNAs (Fig. 3b–d) showed clear separation of the groups compared in each category, for all categories.

**Fig. 4:**
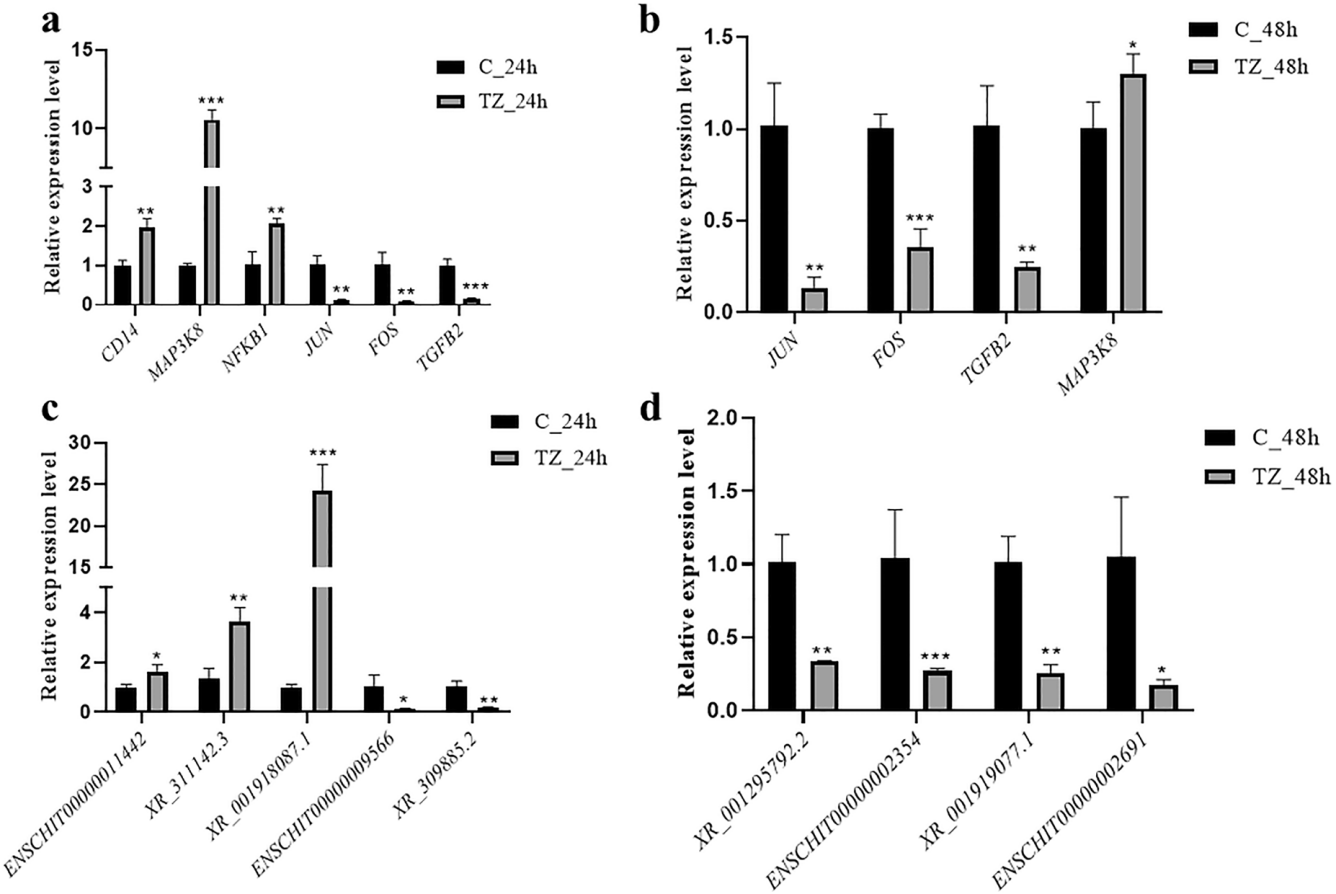
Validation of the differentially expressed lncRNAs (DElncRNAs) and differentially expressed messenger RNAs (DEmRNAs) using quantitative real-time PCR (qRT-PCR). **a, b** The validation result for mRNAs in the categories TZ_24h-vs-C_24h (**a**) and TZ_48h-vs-C_48h (**b**). **c, d** The validation result for lncRNAs in the categories TZ_24h-vs-C_24h (**c**) and TZ_48h-vs-C_48h (**d**). *P < 0.05, **P < 0.01, ***P < 0.001

To verify the reliability of RNA-seq data, six (3 upregulated and 3 downregulated) mRNAs and nine (3 upregulated, 6 downregulated) lncRNAs were randomly selected for qRT-PCR analysis. The expression levels of *cluster of differentiation 14* (*CD14*), *mitogen-activated protein kinase kinase kinase 8* (*MAP3K8*) and *nuclear factor κB subunit 1* (*NFKB1*), and lncRNAs *ENSCHIT00000011442*, *XR_311142.3* and *XR_001918087.1* were increased, while the expression levels of *Jun proto-oncogene* (*JUN*), *proto-oncogene c-Fos-like protein* (*FOS*) and *transforming growth factor beta 2* (*TGFB2*) and lncRNAs *ENSCHIT00000009566*, *XR_309885.2*, *XR_001295792.2*, *ENSCHIT00000002354*, *XR_001919077.1* and *ENSCHIT00000002691* were decreased in the experimental groups (Fig. 4), consistent with the transcriptome data, indicating high reproducibility and correctness of the transcriptome data by RNA-seq.

### Functional prediction of the DEmRNAs

**Fig. 5:**
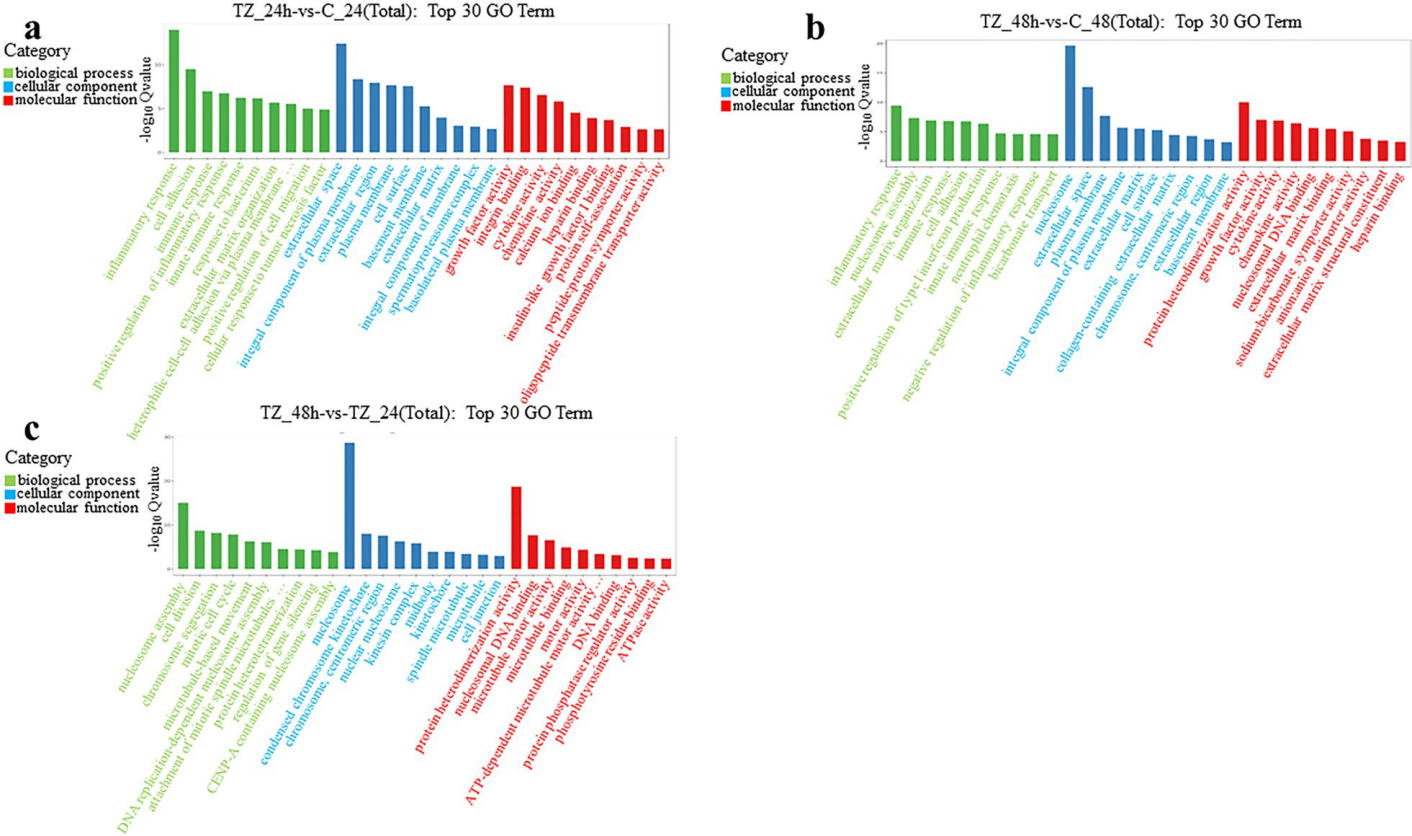
Gene Ontology (GO) enrichment analysis of the differentially expressed messenger RNAs (DEmRNAs) in caprine endometrial epithelial cells (EECs) following *Neospora caninum* infection. **a-c** The top 30 GO terms enriched with DEmRNAs within the categories TZ_24h-vs-C_24h (**a**), TZ_48h-vs-C_48h (**b**) and TZ_48h-vs-TZ_24h (**c**), respectively. A *q*-value < 0.05 and Log2|FC|> 1 were considered to be significant

The GO enrichment analysis of DEmRNAs showed that 2590, 2971 and 1740 terms were significantly enriched within the categories TZ_24h-vs-C_24h, TZ_48h-vs-C_48h and TZ_48h-vs-TZ_24h, respectively (Additional file 6: Data S6). The top 30 most significantly enriched GO terms in cellular component (CC), biological process (BP) and molecular function (MF) are listed in Fig. 5. Of these, inflammatory response (GO: 0006954), extracellular space (GO: 0005615) and growth factor activity (GO: 0008083) in the category TZ_24h-vs-C_24h, inflammatory response (GO: 0006954), nucleosome (GO: 0000786) and protein heterodimerization activity (GO: 0046982) in the category TZ_48h-vs-C_48h and nucleosome assembly (GO: 0006334), nucleosome (GO: 0000786) and protein heterodimerization activity (GO: 0046982) in the category TZ_48h-vs-TZ_24h were most significantly enriched in BP, CC and MF, respectively (Fig. 5).

**Fig. 6:**
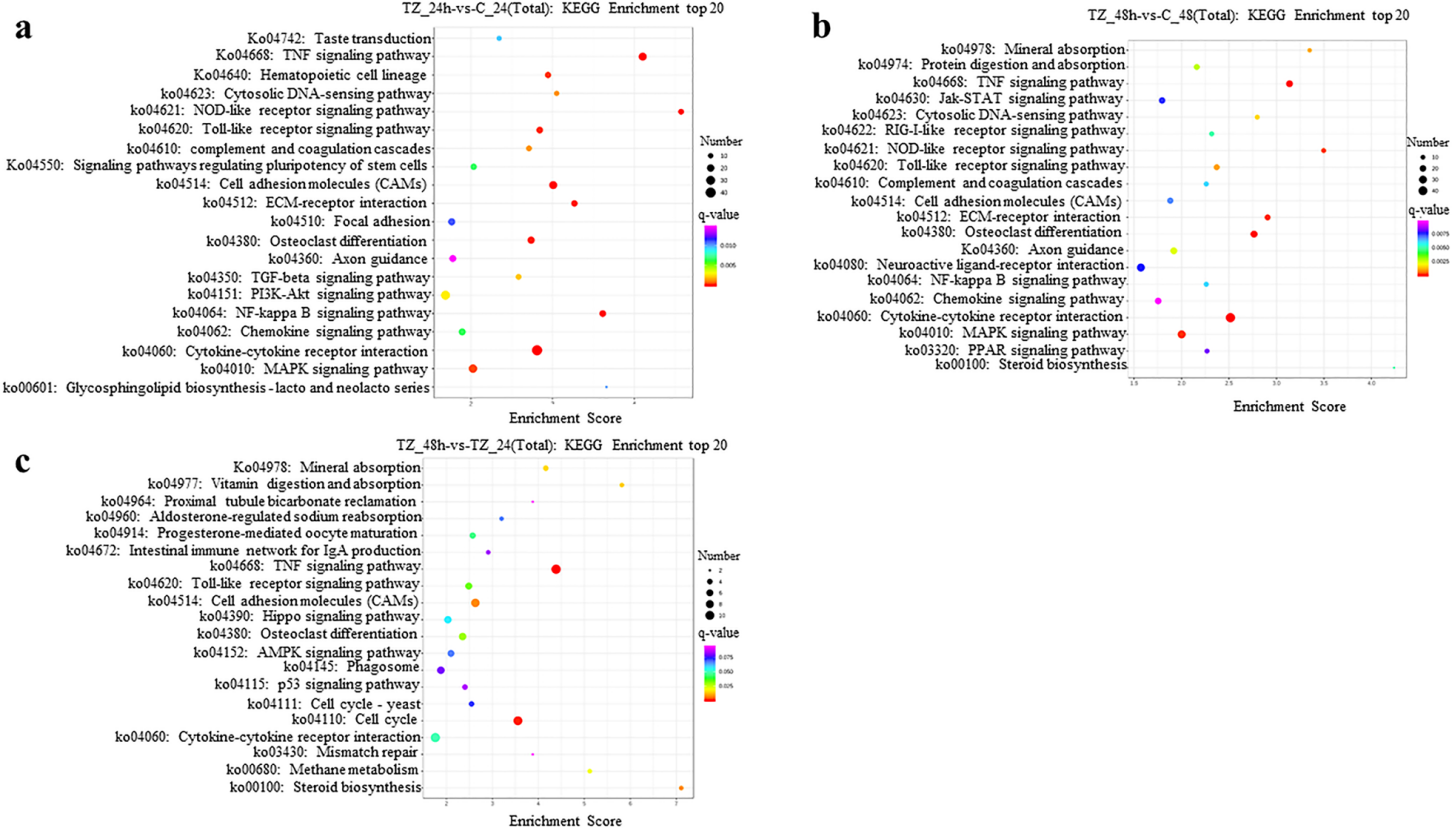
Kyoto Encyclopedia of Genes and Genomes (KEGG) pathway enrichment analysis of the differentially expressed messenger RNAs (DEmRNAs) in caprine endometrial epithelial cells (EECs) following *Neospora caninum* infection. **a**–**c** The top 20 KEGG pathway terms enriched with DEmRNAs within the categories TZ_24h-vs-C_24h (**a**), TZ_48h-vs-C_48h (**b**) and TZ_48h-vs-TZ_24h (**c**), respectively. A *q*-value < 0.05 and Log2|FC|> 1 were considered to be significant

KEGG pathway enrichment analysis showed that 201, 211 and 161 pathways were significantly enriched within the categories TZ_24h-vs-C_24h, TZ_48h-vs-C_48h and TZ_48h-vs-TZ_24h, respectively (Additional file 7: Data S7); the top 20 most significantly enriched pathways are listed in Fig. 6. Of these, signaling molecules and interaction (e.g. cytokine-cytokine receptor interaction, cell adhesion molecules [CAMs] and extracellular matrix [ECM]-receptor interaction), regulation of host immune response (e.g. tumor necrosis factor [TNF] signaling pathway, MAPK signaling pathway, transforming growth factor beta [TGF-beta] signaling pathway, AMPK signaling pathway, Toll-like receptor [TLR] signaling pathway and NOD-like receptor [NLR]) signaling pathway) were main pathways involved (Fig. 6). Interestingly, *N. caninum* infection induced significantly upregulated expressions of several TLR family members (e.g. *TLR2*, *TLR*3 and *TLR*9), NLR family members (e.g. *NOD1* and *NLRP3*), pro-inflammatory cytokines (e.g. *IL1A*, *IL1B*, *IL6*, *IL33* and *IL34*), chemokines (e.g. *CCL20*, *CCL5*, *CXCL16*, *CXCL8* and *CX3CL1*), colony-stimulating factor (CSF) (e.g. *CSF2* and *CSF3*) and TNF receptor superfamily members (e.g. *TNFRSF21*, *TNFSF13B* and *TNFSF15*) in caprine EECs (Additional file 5: Data S5).

### Co-expression analysis and prediction of DElncRNA targets

The co-expression analysis identified 61,001, 99,774 and 9306 DElncRNA-DEmRNA relationships within the categories TZ_24h-vs-C_24h, TZ_48h-vs-C_48h and TZ_48h-vs-TZ_24h, respectively (Additional file 8: Data S8). The gene co-expression networks for *cis*- and *trans*-targets of DElncRNAs are shown in Additional file 9: Fig. S1; Additional file 10: Fig. S2, respectively. Further, 30, 26 and one *cis*-targets of DElncRNAs were respectively co-expressed with 35, 29 and one DElncRNAs, comprising 35, 29 and one relationship within categories TZ_24h-vs-C_24h, TZ_48h-vs-C_48h and TZ_48h-vs-TZ_24h, respectively (Additional file 11: Data S9). 83, 130 and 30 *trans*-targets of DElncRNAs were respectively predicted for 28, 41 and 10 DElncRNAs, comprising 460, 957 and 110 relationships within categories TZ_24h-vs-C_24h, TZ_48h-vs-C_48h and TZ_48h-vs-TZ_24h, respectively (Additional file 12: Data S10).

### Functional analysis of the targets for DElncRNAs

**Fig. 7:**
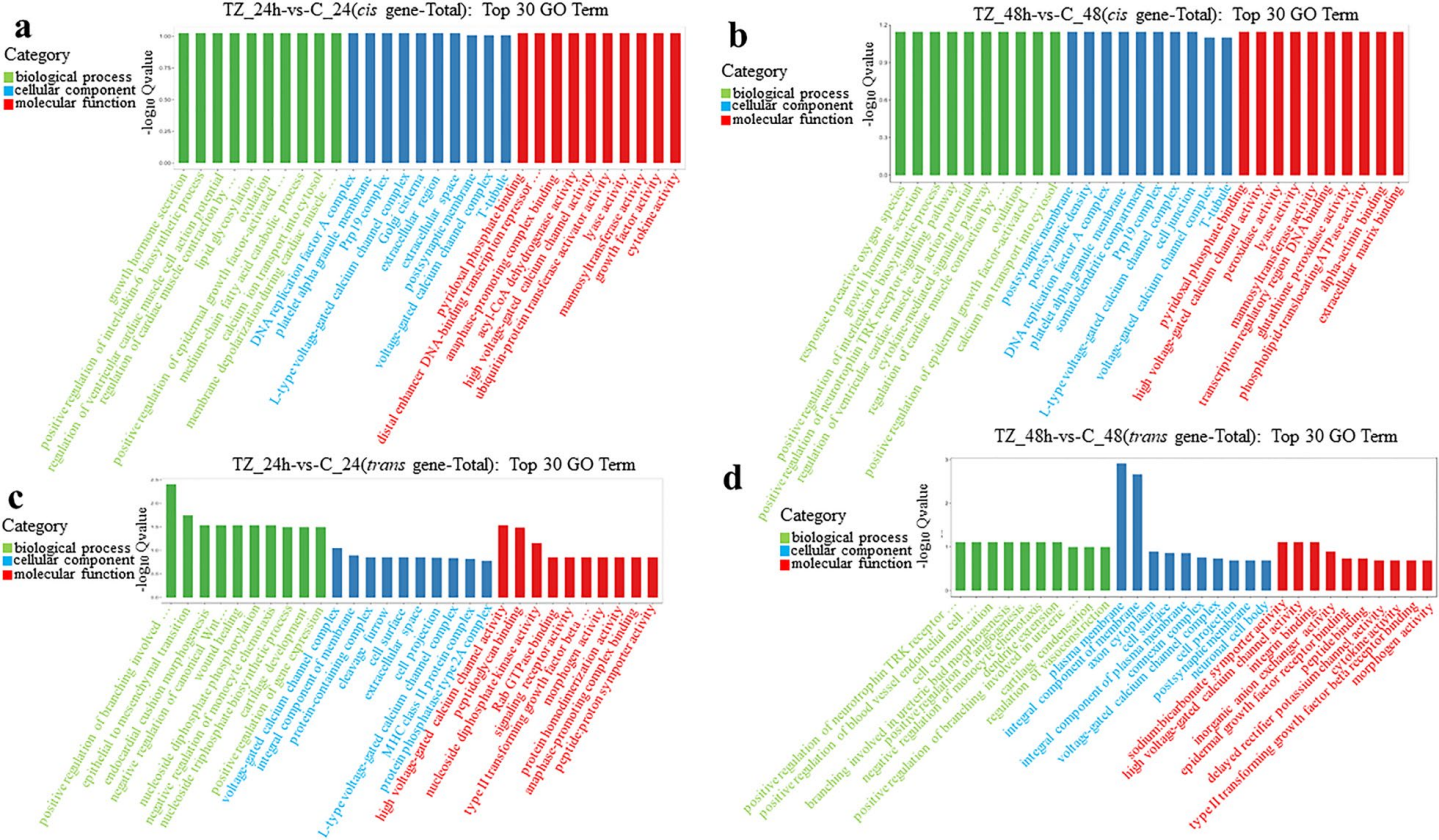
Gene Ontology (GO) enrichment analysis for the *cis*- and *trans*-targets of the differentially expressed lncRNAs (DElncRNAs) in caprine endometrial epithelial cells (EECs) following *Neospora caninum* infection. **a**–**d** The top 30 GO terms enriched for the *cis*- (**a, b**) and *trans*- (**c, d**) targets of DElncRNAs within the categories TZ_24h-vs-C_24h and TZ_48h-vs-C_48h, respectively. A *q*-value < 0.05 and Log2|FC|> 1 were considered to be significant

The GO enrichment analyses of potential *cis*-targets of DElncRNAs showed that 254, 232 and 44 terms were significantly enriched in the categories TZ_24h-vs-C_24h, TZ_48h-vs-C_48h and TZ_48h-vs-TZ_24h, respectively (Additional file 13: Data S11). Further, 534, 701 and 1694 terms of potential *trans*-targets of DElncRNAs were found to be significantly enriched in the categories TZ_24h-vs-C_24h, TZ_48h-vs-C_48h and TZ_48h-vs-TZ_24h, respectively (Additional file 14: Data S12). The top 30 most significantly enriched GO terms of potential *cis*-targets and *trans*-targets of DElncRNAs in BP, CC and MF are listed in Fig. 7 and Additional file 15: Fig. S3.

**Fig. 8:**
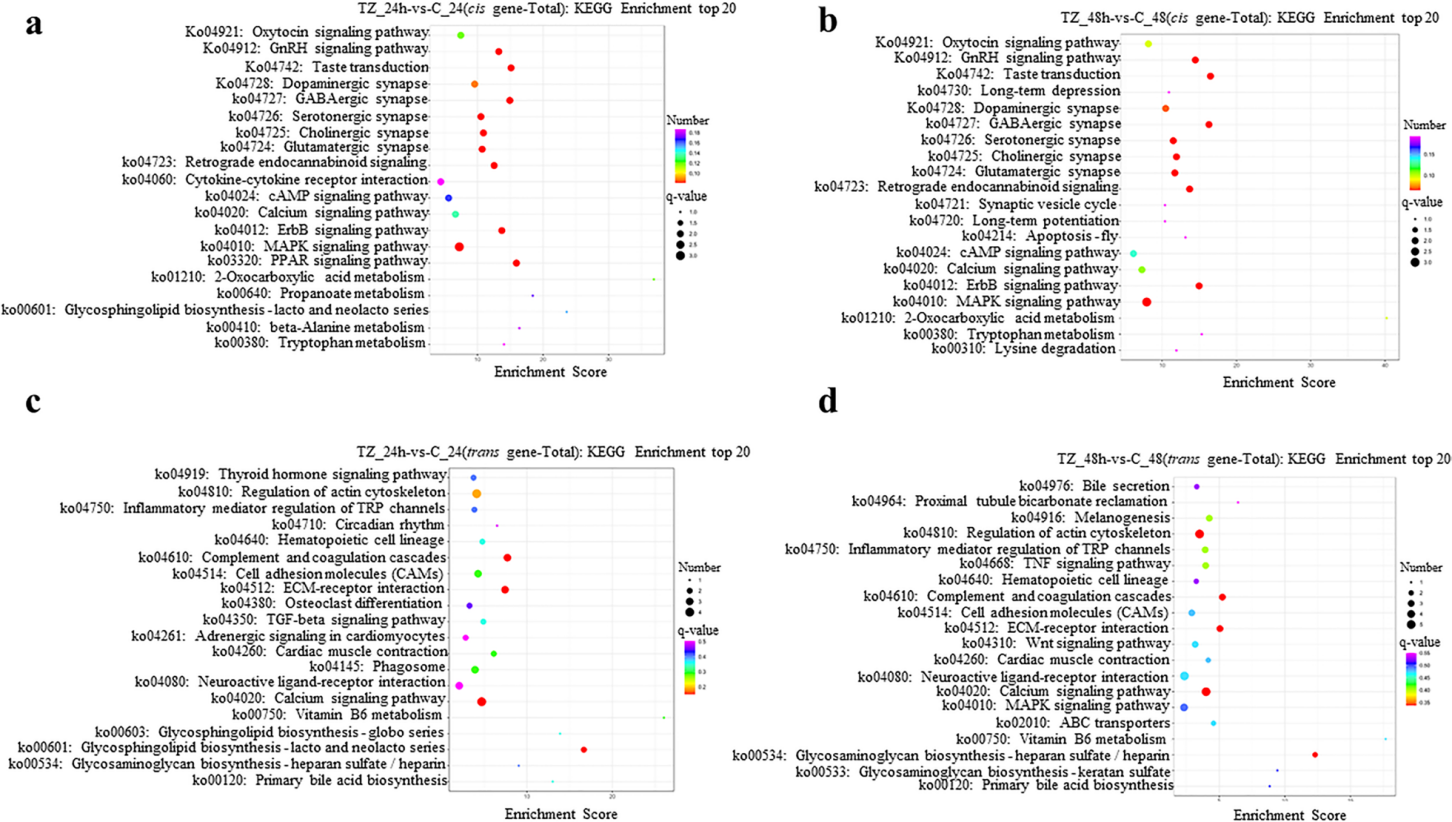
Kyoto Encyclopedia of Genes and Genomes (KEGG) pathway enrichment analysis for the *cis*- and *trans*-targets of the differentially expressed lncRNAs (DElncRNAs) in caprine endometrial epithelial cells (EECs) following *Neospora caninum* infection. **a**–**d** The top 20 KEGG pathway terms enriched for the *cis*- (**a, b**) and *trans*- (**c, d**) targets of DElncRNAs within the categories TZ_24h-vs-C_24h and TZ_48h-vs-C_48h, respectively. *q*-value < 0.05 and Log2|FC|> 1 are considered to be significant

KEGG pathway enrichment analysis of potential *cis*-targets of DElncRNAs showed that 55, 47 and 20 pathways were significantly enriched within the categories TZ_24h-vs-C_24h, TZ_48h-vs-C_48h and TZ_48h-vs-TZ_24h, respectively (Additional file 16: Data S13) and that 62, 80 and 158 pathways of potential *trans*-targets of DElncRNAs were significantly enriched within the categories TZ_24h-vs-C_24h, TZ_48h-vs-C_48h and TZ_48h-vs-TZ_24h, respectively (Additional file 17: Data S14). The top 20 most significantly enriched pathways are listed in Fig. 8 and Additional file 18: Fig. S4. Of these, signal transduction (e.g. MAPK signaling pathway, PPAR signaling pathway, ErbB signaling pathway, calcium signaling pathway, TNF signaling pathway and AMPK signaling pathway), neural transmission (e.g. GABAergic synapse, serotonergic synapse, cholinergic synapse, glutamatergic synapse, dopaminergic synapse, retrograde endocannabinoid signaling), signaling molecules and interaction (e.g. cytokine-cytokine receptor interaction, ECM-receptor interaction and CAMs), and metabolism (e.g. glycosphingolipid biosynthesis-lacto and neolacto series, glycosaminoglycan biosynthesis-heparan sulfate/heparin, 2-Oxocarboxylic acid, propanoate, beta-alanine, tryptophan, vitamin B6, primary bile acid biosynthesis) were main pathways involved in.

## Discussion

The uterus, one of the main reproductive organs needed to maintain normal pregnancy, can be naturally infected by *N. caninum* [32–34]. Previous studies mainly focused on bio-functions of host protein-coding genes [48, 49] or on organelles of *N. caninum* tachyzoites (e.g. microneme, rhoptry and dense granule) [50–52] during *N. caninum* infection, but the role of host lncRNAs during *N. caninum* infection has not been investigated prior to the present study. Recent studies have shown that host lncRNAs play important roles in cellular molecular regulatory networks, and these have been reported to be closely related to the pathogenesis of apicomplexan parasites [53]. For example, genome-wide RNA transcriptome analysis identified 3942 DEmRNAs and 1839 DElncRNAs in murine intestinal epithelial cells following *Cryptosporidium parvum* infection. Of these, a lncRNA, named *NR_045064*, could reduce infection burden of parasites in murine intestinal epithelial cells in vitro and in the enteroids of neonatal mice by promoting expression of host defense genes (e.g. *Csf2*, *Nos2*, and *Cxcl2*) [54]. Similarly, a total of 109 DEmRNAs and 996 DElncRNAs were identified in human foreskin fibroblast (HFF) cells infected with *Toxoplasma gondii* by using microarray, and a novel lncRNA, named *NONSHAT022487* was found to be able to decrease the expression of several host cytokines (e.g. *IFN-γ*, *TNF-α*, *IL-1β* and *IL-12*) by negatively regulating the immune-related molecule *UNC93B1* [55]. In the present study, we found that *N. caninum* infection significantly altered the expression of mRNAs and lncRNAs in caprine EECs at 24 and 48 hpi.

Pattern recognition receptors (PRRs) are pivotal parts of host innate immunity that recognize specific pathogen-associated molecular patterns (PAMPs) to induce secretion of inflammatory cytokines and chemokines through initiating intracellular signaling cascades, ultimately eliminating invading pathogens and infected cells [56]. These PRRs include TLRs, NLRs, RIG-I-like receptors (RLRs) and C-type lectin receptors (CLRs). Among them, TLRs and NLRs play important roles in mediating host innate and adaptive immune responses against *N. caninum* infection [48, 57, 58]. The expression of *TLR2* was efficiently activated in immune cells (e.g. bovine/mouse peritoneal macrophage cell) infected with *N. caninum* or treated with its derived antigens (e.g. glycosylphosphatidylinositol [GPI], extracellular vesicles [EVs], soluble antigens, *N. caninum* cyclophilin [NcCyp]) and could remarkably enhance production of Th1 immune responses, which is critical for controlling *N. caninum* infection. *TLR2* knockout (*TLR2*^−/−^) mice displayed higher parasite loads than wild-type mice [58–62]. *TLR3* was also activated in murine macrophages infected with *N. caninum*, and it could enhance expression of the type I interferon (*IFN-α* and *IFN-β*) by initiating adaptor protein TRIF. *TLR3* knockout (*TLR3*^−/−^) mice reduced the survival rates of infected mice [57]. Furthermore, in non-professional immune cells, the expression of *TLR2* was also induced in both bovine trophoblast cells and caruncular cells infected with *N. caninum* [63], and *TLR3*, *7*, *8* and *9* were upregulated in the maternal–fetal interface in cattle infected with *N. caninum* or immunized with soluble whole antigens or recombinant *N. caninum* proteins [64, 65]. In our study, *TLR2*, *TLR3* and *TLR9* were significantly upregulated in caprine EECs infected with *N. caninum*, consistent with gene expression profiling in boMØs infected with *N. caninum* [13]. In addition, *NLRP3* inflammasome and *NOD1* were also activated in caprine EECs infected with *N. caninum*. Previous studies showed that *N. caninum* infection activated the *NLRP3* inflammasome in murine bone marrow-derived macrophages or bovine peritoneal macrophage cells, accompanied by cleavage of *caspase-1*, release of *IL-1β* and *IL-18*, as well as cell death against *N. caninum* infection, and *NLRP3* knockout (*NLRP3*^−/−^) mice displayed a high susceptibility to *N. caninum* infection [66, 67]. Although the role of *NOD1* in host cells infected with *N. caninum* remains unknown, *NOD1* can mediate host defenses against bacterial, viral and other parasitic infections [68]. These findings suggest that activation of TLRs and NLRs in caprine EECs during *N. caninum* would trigger protective innate defense mechanisms against *N. caninum* infection.

Cytokines are major messenger proteins of inflammatory process and immune responses with various biological effects (e.g. cell growth, differentiation, inflammatory response and immune defense) [69]. Previous studies have confirmed that pro-inflammatory cytokines (e.g. *IFN-γ*, *TNF-α*, *IL-12*, *IL6* and *IL1β*) induced by *N. caninum* infection could exert protective immunity to inhibit the multiplication of *N. caninum* both in vitro [70, 71] and in vivo [59, 72]. In our study, a large number of pro-inflammatory cytokines (e.g. *IL1A*, *IL1B*, *IL6*, *IL33* and *IL34*) were up-regulated, and the anti-inflammatory cytokines (e.g. *TGFB2* and *TGFB3*) was down-regulated, suggesting that *N. caninum* infection promoted the secretion of pro-inflammatory cytokines in caprine EECs. In addition, chemokines (e.g. *CCL20*, *CCL5*, CXCL16, *CXCL8* and *CX3CL1*), colony-stimulating factor (e.g. *CSF2* and *CSF3*) and tumor necrosis factor receptor superfamily member (e.g. *TNFRSF21*, *TNFSF13B* and *TNFSF15*) involved in immune modulatory properties were also upregulated in caprine EECs during *N. caninum* infection. These data suggest that cytokines induced by *N. caninum* infection in caprine EECs would be a strategy for eliciting immune responses at the maternal–fetal interface against *N. caninum* infection, but the disruption of the immune balance at the maternal–fetal interface was also reported to lead to miscarriage [73, 74].

To understand the potential regulatory functions of DElncRNAs in caprine EECs during *N. caninum* infection, we predicted the *cis*- and *trans*-targets of DElncRNAs by constructing lncRNA-mRNA co-expression networks. We found that numerous biological signal pathways were significantly enriched, including MAPK signaling pathway, PPAR signaling pathway, ErbB signaling pathway, calcium signaling pathway, TNF signaling pathway and AMPK signaling pathway. These pathways have been reported to be involved in the regulation of important biological processes, such as cell proliferation, apoptosis, autophagy, inflammatory and immune response [75–80]. For example, upregulated expressions of lncRNA* XR_001296952.2* and lncRNA * XR_001919803.1* were found to *cis*-regulate the expression of *stanniocalcin‑2* (*STC2*), which could promote proliferation and inhibit apoptosis in caprine EECs through the RAS/RAF/MEK/ERK signaling pathways [81], and could also enhance autophagy through the PI3K/AKT/AMPK signaling pathways [82]. Previous studies have reported that activation of autophagy facilitated the proliferation of *N. caninum* both in vitro [35] and in vivo [83]. In bone marrow-derived macrophages, the activation of p38 MAPK was found to be associated with immune evasion of *N. caninum* [84]. However, in Madin-Darby bovine kidney (MDBK) cells, p38 MAPK inhibitor effectively inhibited *N. caninum* tachyzoite motility and micronemal protein secretion and reduced cell invasion of *N. caninum* [85]. In addition, neural transmission (e.g. GABAergic synapse, serotonergic synapse, cholinergic synapse, glutamatergic synapse, dopaminergic synapse and retrograde endocannabinoid signaling), metabolism processes (e.g. glycosphingolipid biosynthesis-lacto and neolacto series, glycosaminoglycan biosynthesis-heparan sulfate/heparin, 2-oxocarboxylic acid, propanoate, beta-alanine, tryptophan, vitamin B6 and primary bile acid biosynthesis) and signaling molecules and interaction (e.g. cytokine-cytokine receptor interaction, ECM-receptor interaction and CAMs) were also significantly enriched for DElncRNA targets, indicating that these DElncRNAs would play roles in regulating host neural transmission, metabolism processes and interactions between signaling molecules during *N. caninum* infection. Both ECM-receptor interaction and CAMs are associated with endometrial receptivity, the key to successful implantation and development of mammalian embryos [86, 87], suggesting that these lncRNA targets would influence the outcome of pregnancy during *N. caninum* infection.

## Conclusions

*Neospora caninum* infection significantly altered the expression profiles of mRNAs and lncRNAs in caprine EECs at 24 and 48 hpi. The identified DEmRNAs and DElncRNAs were involved in immune response, signal transduction, nervous and metabolic processes during *N. caninum* infection. To our knowledge, this is the first investigation of global profiles of host lncRNAs during *N. caninum* infection. The results provide novel insight into understanding the underlying pathogenesis of *N. caninum* in maternal–fetal interface. However, since functions of most goat lncRNAs identified in our study are still unknown, computerized prediction of their function is very difficult if not completely impossible. Therefore, further studies should be conducted to reveal the mysterious veil of these identified DElncRNAs in future studies.

## Supplementary Information

**Additional file 1: Data S1**. The primer sequences used in this study by quantitative real-time PCR (qRT‑PCR).

**Additional file 2: Data S2**. The statistics of sequencing data.

**Additional file 3: Data S3**. Length, exon numbers and classification of the long noncoding RNAs (lncRNAs).

**Additional file 4: Data S4**. All differentially expressed lncRNAs (DElncRNAs).

**Additional file 5: Data S5**. All differentially expressed mRNAs (DEmRNAs).

**Additional file 6: Data S6**. Gene Ontology (GO) enrichment analysis of all differentially expressed mRNAs (DEmRNAs).

**Additional file 7: Data S7**. Kyoto Encyclopedia of Genes and Genomes (KEGG) pathway analysis of all differentially expressed mRNAs (DEmRNAs).

**Additional file 8: Data S8**. The gene co-expression analysis of differentially expressed (DE) lncRNAs and DEmRNAs.

**Additional file 9: Figure S1**. The gene co-expression networks of* cis*-targets of differentially expressed lncRNAs (DElncRNAs) in caprine endometrial epithelial cells (EECs) following* Neospora caninum* infection.** a**-**c** The top 20 most significantly enriched *cis*-targets of DElncRNAs within the categories TZ_24h-vs-C_24h (**a**), TZ_48h-vs-C_48h (**b**), and TZ_48h-vs-TZ_24h (**c**), respectively. The left and right sides of the* y*-axis represent mRNA and lncRNA, respectively, and the* x*-axis represents the distance between mRNA and lncRNA, with negative values representing upstream and positive values representing downstream. *P< 0.05, **P< 0.01, ***P< 0.001.

**Additional file 10: Figure S2**. The gene co-expression networks of *trans*-targets of differentially expressed lncRNAs (DElncRNAs) in caprine endometrial epithelial cells (EECs) following *Neospora caninum* infection.** a**-**c** Co-repression network of DElncRNAs with their *trans*-targets within the categories TZ_24h-vs-C_24h (**a**), TZ_48h-vs-C_48h (**b**), and TZ_48h-vs-TZ_24h (**c**), respectively. The red nodes represent lncRNAs, the green nodes represent mRNAs, and the node size represents the number of genes.

**Additional file 11: Data S9**. The* cis*-target genes predicted for differentially expressed lncRNAs (DElncRNAs).

**Additional file 12: Data S10**. The* trans*-target genes predicted for differentially expressed lncRNAs (DElncRNAs).

**Additional file 13: Data S11**. Gene Ontology (GO) enrichment analysis of all* cis*-target genes for the differentially expressed lncRNAs (DElncRNAs).

**Additional file 14: Data S12**. Gene Ontology (GO) enrichment analysis of all* trans*-target genes for the differentially expressed lncRNAs (DElncRNAs).

**Additional file 15: Figure S3**. Gene Ontology (GO) enrichment analysis for the *cis*- and *trans*-targets of the differentially expressed lncRNAs (DElncRNAs) in caprine endometrial epithelial cells (EECs) following* Neospora caninum* infection.** a**,** b** The top 30 GO terms enriched for the *cis*- (**a**) and* trans*- (**b**) targets of DElncRNAs within the category TZ_48h-vs-TZ_24h. A* q*-value < 0.05 and Log2|FC| > 1 are considered to be significant.

**Additional file 16: Data S13**. Kyoto Encyclopedia of Genes and Genomes (KEGG) pathway enrichment analysis of all* cis*-target genes for the differentially expressed lncRNAs (DElncRNAs).

**Additional file 17: Data S14**. Kyoto Encyclopedia of Genes and Genomes (KEGG) pathway enrichment analysis of all* trans*-target genes for the differentially expressed lncRNAs (DElncRNAs).

**Additional file 18: Figure S4**. Kyoto Encyclopedia of Genes and Genomes (KEGG) pathway enrichment analysis for the *cis*- and *trans*-targets of the differentially expressed lncRNAs (DElncRNAs) in caprine endometrial epithelial cells (EECs) following* Neospora caninum *infection., The top 20 KEGG pathway terms enriched for the* cis*- (a) and* trans*- (b) targets of DElncRNAs within the category TZ_48h-vs-TZ_24h. A* q*-value < 0.05 and Log2|FC| > 1 are considered to be significant.

## Data Availability

The datasets supporting the findings of this article are included within the article and its additional files. The original data were deposited in the NCBI repository under accession number is PRJNA838937.

## Abbreviations

boMØs: Bovine monocyte-derived macrophages
BP: Biological process
CAMs: Cell adhesion molecules
CC: Cellular component
CNCI: Coding-noncoding index
CPC2: Coding potential calculator 2
DE: Differentially expressed
DElncRNAs: Differentially expressed lncRNAs
DEmRNAs: Differentially expressed mRNAs
EECs: Endometrial epithelial cells
FC: Fold change
GO: Gene Ontology
hpi: Hours post-infection
KEGG: Kyoto Encyclopedia of Genes and Genomes
lncRNAs: Long noncoding RNAs
MF: Molecular function
NLR: NOD-like receptor
mRNAs: Messenger RNAs
PLEK: Predictor of long-noncoding RNAs and messenger RNAs based on an improved *k*-mer scheme
PRRs: Pattern recognition receptors
qRT-PCR: Quantitative real-time PCR
RNA-seq: RNA sequencing
*TGFB2*: *Transforming growth factor beta* *2*
TLRs: Toll-like receptors
*TNF*: *Tumor necrosis factor*
