# Supplementary figures and images for: RNA sequencing reveals dynamic expression of lncRNAs and mRNAs in caprine endometrial epithelial cells induced by Neospora caninum infection
Source: Parasit Vectors. 2022 Aug 24;15:297. doi: 10.1186/s13071-022-05405-5 (PMC9398501; doi:10.1186/s13071-022-05405-5)

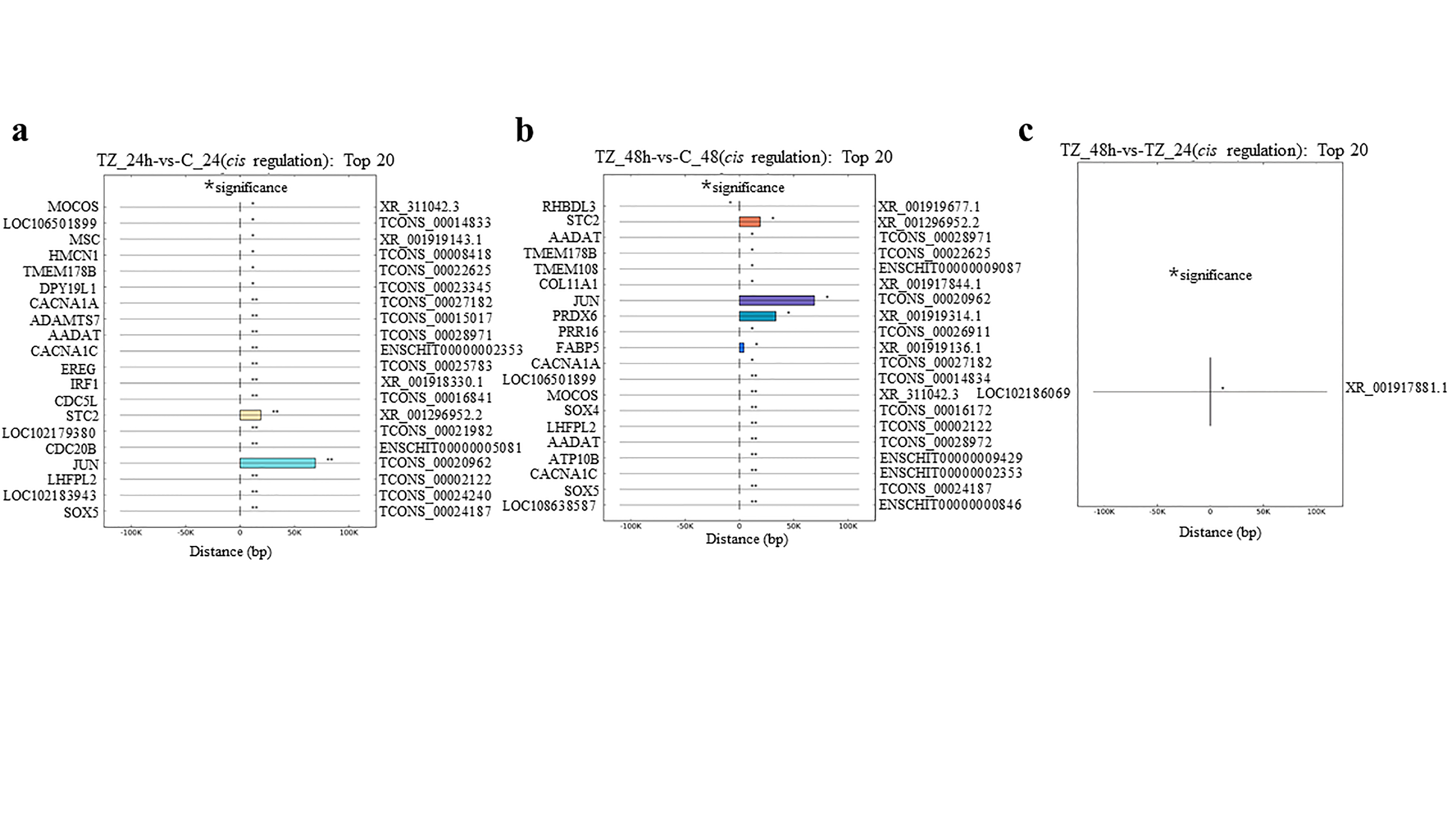

Supplement: Supplementary file 9 — Additional file 9: Figure S1. The gene co-expression networks of cis-targets of differentially expressed lncRNAs (DElncRNAs) in caprine endometrial epithelial cells (EECs) following Neospora caninum infection. a-c The top 20 most significantly enriched cis-targets of DElncRNAs within the categories TZ_24h-vs-C_24h (a), TZ_48h-vs-C_48h (b), and TZ_48h-vs-TZ_24h (c), respectively. The left and right sides of the y-axis represent mRNA and lncRNA, respectively, and the x-axis represents the distance between mRNA and lncRNA, with negative values representing upstream and positive values representing downstream. *P< 0.05, **P< 0.01, ***P< 0.001. [file 13071_2022_5405_MOESM9_ESM.tif]

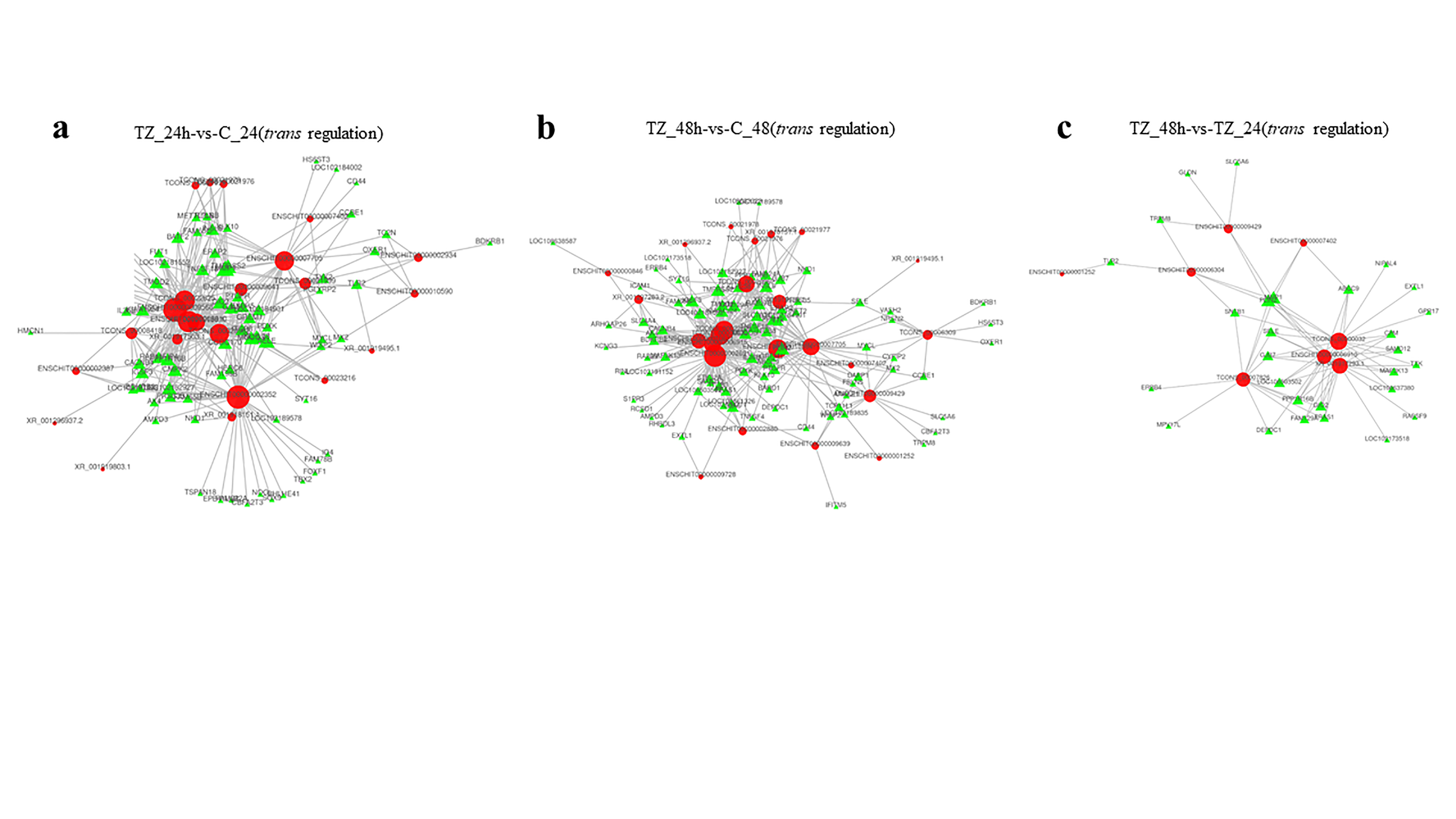

Supplement: Supplementary file 10 — Additional file 10: Figure S2. The gene co-expression networks of trans-targets of differentially expressed lncRNAs (DElncRNAs) in caprine endometrial epithelial cells (EECs) following Neospora caninum infection. a-c Co-repression network of DElncRNAs with their trans-targets within the categories TZ_24h-vs-C_24h (a), TZ_48h-vs-C_48h (b), and TZ_48h-vs-TZ_24h (c), respectively. The red nodes represent lncRNAs, the green nodes represent mRNAs, and the node size represents the number of genes. [file 13071_2022_5405_MOESM10_ESM.tif]

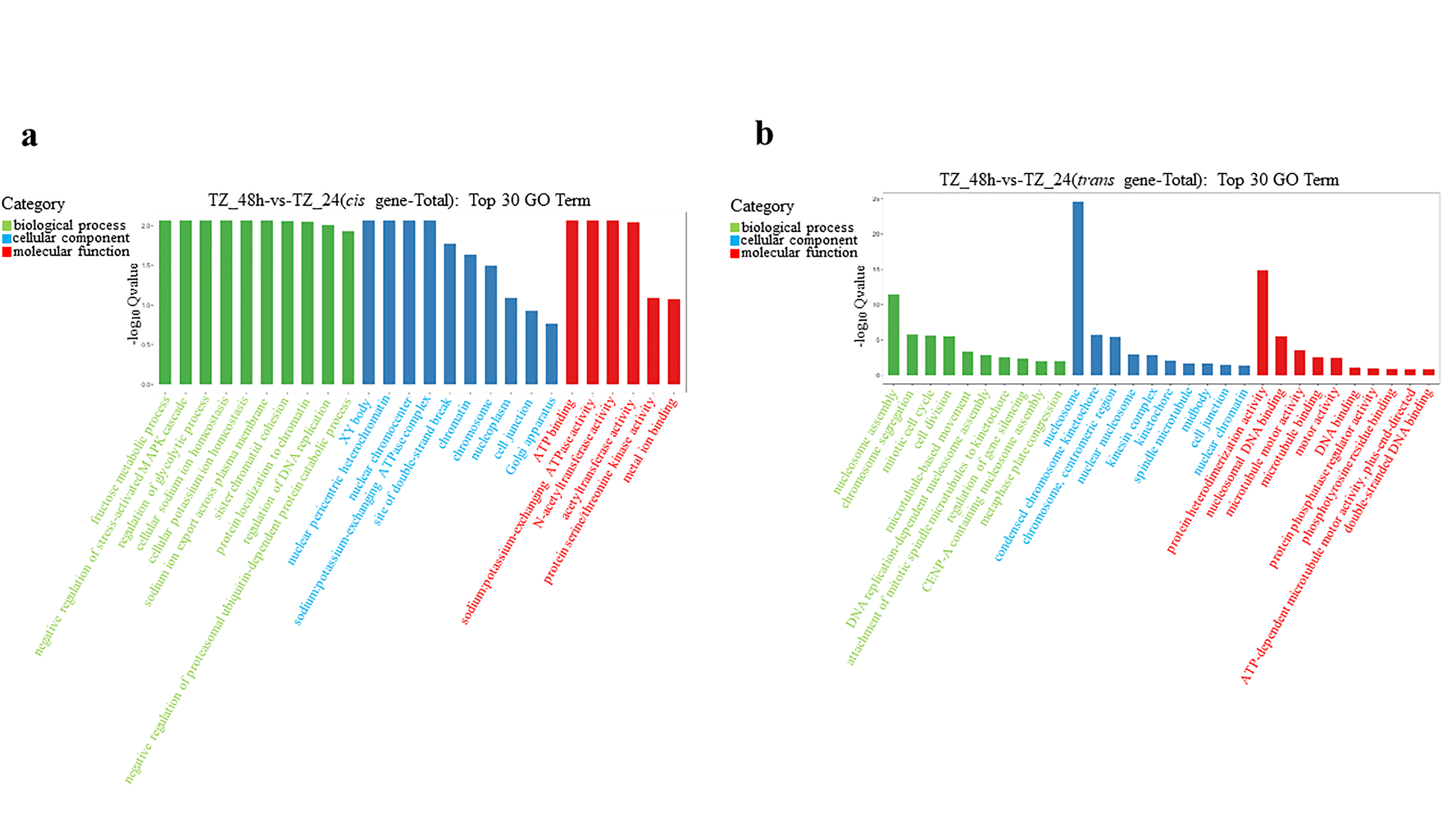

Supplement: Supplementary file 15 — Additional file 15: Figure S3. Gene Ontology (GO) enrichment analysis for the cis- and trans-targets of the differentially expressed lncRNAs (DElncRNAs) in caprine endometrial epithelial cells (EECs) following Neospora caninum infection. a, b The top 30 GO terms enriched for the cis- (a) and trans- (b) targets of DElncRNAs within the category TZ_48h-vs-TZ_24h. A q-value < 0.05 and Log2|FC| > 1 are considered to be significant. [file 13071_2022_5405_MOESM15_ESM.tif]

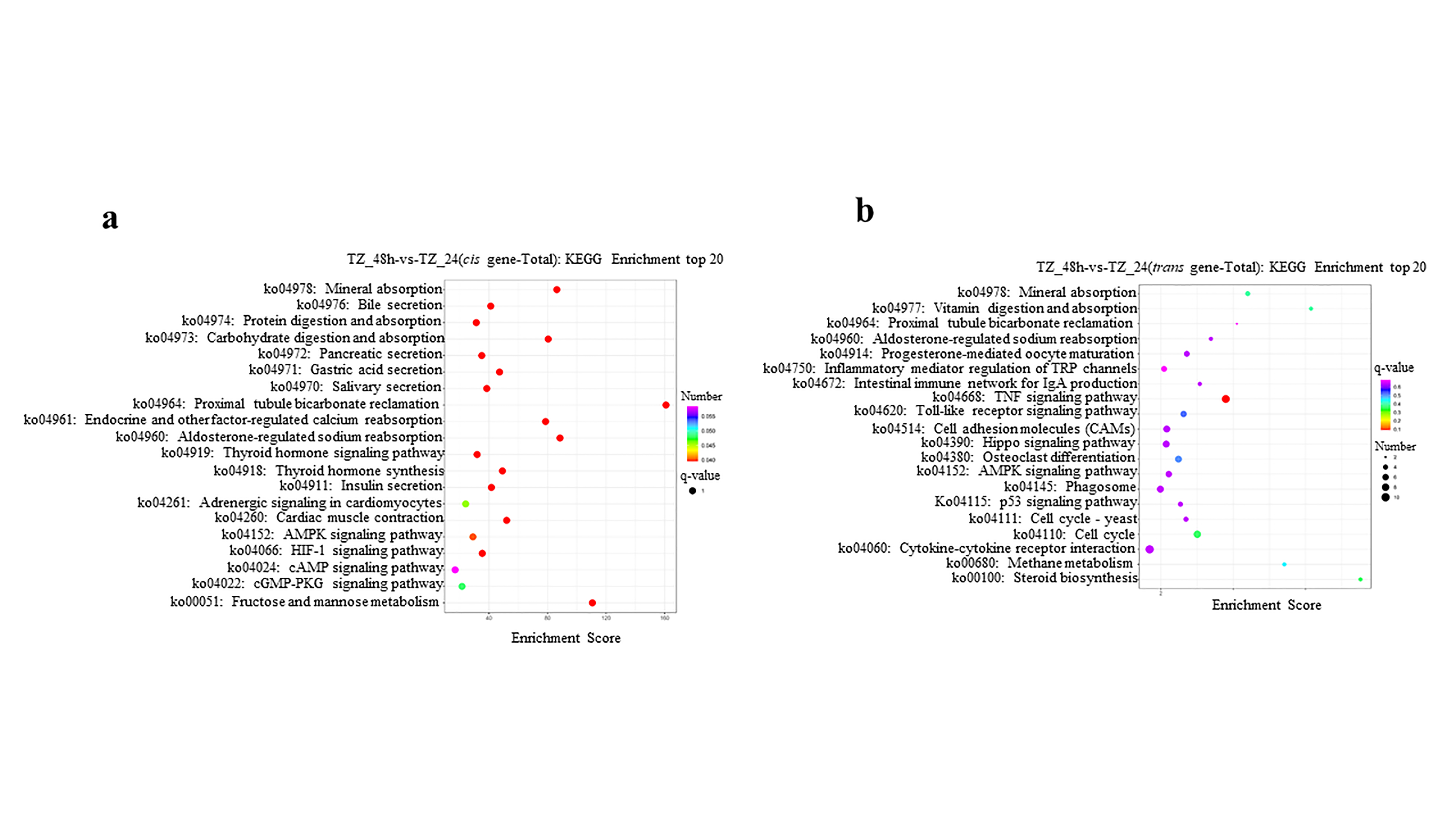

Supplement: Supplementary file 18 — Additional file 18: Figure S4. Kyoto Encyclopedia of Genes and Genomes (KEGG) pathway enrichment analysis for the cis- and trans-targets of the differentially expressed lncRNAs (DElncRNAs) in caprine endometrial epithelial cells (EECs) following Neospora caninum infection., The top 20 KEGG pathway terms enriched for the cis- (a) and trans- (b) targets of DElncRNAs within the category TZ_48h-vs-TZ_24h. A q-value < 0.05 and Log2|FC| > 1 are considered to be significant. [file 13071_2022_5405_MOESM18_ESM.tif]
